# Psychopathological features of irritable bowel syndrome patients with and without functional dyspepsia: a cross sectional study

**DOI:** 10.1186/1471-230X-11-94

**Published:** 2011-08-26

**Authors:** Daria Piacentino, Rosanna Cantarini, Marianna Alfonsi, Danilo Badiali, Nadia Pallotta, Massimo Biondi, Enrico S Corazziari

**Affiliations:** 1 Department of Neurology and Psychiatry, Sapienza University, Rome, Italy; 2 Department of Internal Medicine and Medical Specialties, Sapienza University, Rome, Italy

## Abstract

**Background:**

Irritable bowel syndrome (IBS) and functional dyspepsia (FD) show considerable overlap and are both associated with psychiatric comorbidity. The present study aimed to investigate whether IBS patients with FD show higher levels of psychopathology than those without FD. As a preliminary analysis, it also evaluated the psychopathological differences, if any, between IBS patients featuring the two Rome III-defined FD subtypes, i.e. postprandial distress syndrome (PDS) and epigastric pain syndrome (EPS).

**Methods:**

Consecutive outpatients (n = 82, F = 67, mean age 41.6 ± 12.7 years) referred to our third level gastroenterological centre, matching the Rome III criteria for IBS and, if present, for concurrent FD, were recruited. They were asked to complete a 90-item self-rating questionnaire, the Symptom Checklist 90 Revised (SCL-90-R), in order to assess the psychological status. Comparisons between groups were carried out using the non-parametric Mann-Whitney U test.

**Results:**

Patients with IBS only were 56 (68.3%, F = 43, mean age 41.6 ± 13.3 years) and patients with both IBS and FD were 26 (31.7%, F = 24, mean age 41.8 ± 11.5 years), 17 of whom had PDS and 9 EPS. Patients with both IBS and FD scored significantly higher on the SCL-90-R GSI and on eight out of the nine subscales than patients with IBS only (*P *ranging from 0.000 to 0.03). No difference was found between IBS patients with PDS and IBS patients with EPS (*P *ranging from 0.07 to 0.97), but this result has to be considered provisional, given the small sample size of the two subgroups.

**Conclusions:**

IBS-FD overlap is associated with an increased severity of psychopathological features. This finding suggests that a substantial subset of patients of a third level gastroenterological centre with both IBS and FD may benefit from psychological assessment and treatment.

## Background

Irritable bowel syndrome (IBS) and functional dyspepsia (FD) are functional gastrointestinal disorders commonly seen in both primary healthcare and gastroenterology practice; they show a prevalence of, respectively, 10%-20% [1] and 20%-30% [2] in the general population.

Recent epidemiological studies, conducted on the general population as well as on patient-based series, demonstrate considerable overlap between IBS and FD [3-5]: a high percentage of IBS patients show coexisting upper gastrointestinal symptoms consistent with FD (epigastric pain, epigastric burning, postprandial fullness, early satiation), and a high percentage of FD patients complain of lower abdominal pain and disturbed bowel habits (diarrhea, constipation, mixed diarrhea and constipation).

In both IBS and FD there is evidence of an association with psychological factors, and comorbidity with psychiatric disorders, especially depression, anxiety and somatization, is high [6,7]. It is unclear whether the co-occurrence of IBS and FD impairs the psychological status to a greater degree than the occurrence of IBS or FD alone. A number of studies support this hypothesis, having found that the presence of upper gastrointestinal symptoms in IBS patients increases the incidence and severity of psychopathology [8,9]. However, a recent study seems to contradict these findings [10]. Given that psychopathological features influence symptom perception, health-care seeking behavior and quality of life in IBS patients [7,11], and that the presence of dyspeptic symptoms, in addition to the IBS ones, may have a negative impact on psychopathological features, it seems worthwhile to comparatively assess these features in IBS patients with and without FD.

It is also unknown whether, in IBS patients with FD, psychopathological features differ between patients with the two subtypes of FD as defined by the Rome III criteria: postprandial distress syndrome (PDS) and epigastric pain syndrome (EPS) [2]. To our knowledge, no study on the relationship between the subtype of FD in IBS patients and psychiatric comorbidity has yet been reported. Since PDS and EPS may have distinct underlying pathophysiology [2,12], it seems of some interest to investigate whether the two FD subtypes also present distinct psychopathological features, thus determining different loads of psychiatric comorbidity in IBS patients.

The aim of the present study was to determine whether IBS patients with FD show higher levels of psychopathology than those without FD. As a preliminary analysis, the study also evaluated whether, in IBS patients with FD, the two Rome III-defined subtypes of FD are associated with different psychopathological features.

## Methods

### Patients

Consecutive IBS outpatients (n = 82, F = 67, mean age 41.6 ± 12.7 years), referred to our third level gastroenterological centre, were recruited. The majority of patients (54.9%) were referred from primary care, whereas the remainder were either self-referred (34.1%) or referred from other gastroenterologists (11.0%). Patients were interviewed by an experienced gastroenterologist. Age, gender, medical history, including gastrointestinal and extra-gastrointestinal complaints, and family history were assessed. The diagnoses of IBS and, if present, of concurrent FD, were made on the basis of the Rome III criteria [1,2] by means of a clinical interview and of a gastrointestinal symptom questionnaire [13]. IBS patients with FD were classified according to their symptoms as having one of the two subtypes of FD, PDS or EPS. All patients had normal serum biochemistry, complete blood count and no significant pathological findings at esophagogastroduodenoscopy and colonoscopy. Patients with another coexisting disease (e.g., malignancy; cardiovascular, pulmonary, hepatic or renal disorder) were excluded. Patients meeting enrollment criteria were asked to fill out a self-report questionnaire, the Symptom Checklist 90 Revised (SCL-90-R) [14], in order to assess psychopathological features. The questionnaire was reviewed with each participant to guarantee that they understood how to complete it. Questionnaires were completed anonymously; a numeric code was assigned to each patient and then entered in a database for statistical analysis. The aim of the study was thoroughly explained to the patients, which gave their written consent to participate.

### Questionnaires

#### Gastrointestinal symptom questionnaire

Gastrointestinal symptoms were investigated by means of the Italian version of the Rome II Modular Questionnaire [13], consisting of 38 items specifically designed to establish the presence of functional gastrointestinal disorders; in the present study we focused on the questions related to the presence of IBS and FD. The questionnaire has been validated and approved for use in the Italian language by the Multinational Working Team to Develop Criteria for Functional Gastrointestinal Disorders and takes approximately 15 minutes to be completed. Although the Rome II Questionnaire was the only one available at the time the study started, since an Italian version of the Rome III Questionnaire has not yet been validated, its items were applicable to both the Rome II and the Rome III IBS and FD criteria. Therefore, it was possible to diagnose IBS on the basis of the Rome III criteria as follows: abdominal pain or discomfort at least 2 days a week for the previous 3 months, accompanied by at least 2 of the following features: (i) it improved with defecation, (ii) it was associated with a change in the frequency of bowel movements, (iii) it was associated with a change in the appearance of the stool. The onset of symptoms had to be at least 6 months prior to the enrollment. Likewise, FD was identified in IBS patients if there was at least one of these symptoms for the previous 3 months: (i) bothersome postprandial fullness, (ii) early satiation, (iii) epigastric pain, (iv) epigastric burning. Symptoms had to start at least 6 months earlier and there had to be no evidence of a structural disease that was likely to explain them. FD as defined above was subdivided, following the Rome III definition, into: 1) postprandial distress syndrome (PDS), consisting of bothersome postprandial fullness (occurring after an ordinary-sized meal), and/or early satiation (preventing the patient to finish the meal); 2) epigastric pain syndrome (EPS), consisting of intermittent pain or burning localized in the epigastric area (i.e. not generalized or localized in other abdominal or chest regions), not relieved by defecation or passage of flatus and not fulfilling the criteria for gallbladder or sphincter of Oddi disorders. No FD patient was defined as the individual not reporting any type of dyspeptic symptoms.

#### Symptom Checklist 90 Revised

Psychopathological features were assessed with the Symptom Checklist 90 Revised (SCL-90-R) [14], a self-administered questionnaire used to evaluate the symptoms of psychopathology experienced by individuals even beyond clinically relevant mental disorders. The questionnaire is appropriate for use in both normal and distressed individuals (i.e. individuals with medical or psychiatric disorders) and has shown good internal consistency, as well as good inter-rater and test-retest reliability [14]. Furthermore, it has already been used in studies on IBS and FD patients, and has been translated and employed extensively in the Italian population [15]. Its administration time is approximately 15 minutes.

The questionnaire consists of 90 items concerning an individual's symptom distress in the previous 7 days and the individual has to assign a score from 0 to 4 depending on the degree of suffering related to the item: consequently, each item is rated on a five-point Likert scale (0-4) from "not at all" to "extremely" distressing. In clinical practice, the SCL-90-R is used to reflect a general symptom level of the individual, i.e. the global severity index (GSI), as well as a more differentiated subscale profile. The nine subscales that can be derived from the SCL-90-R are: somatization (SOM, 12 items), obsessive-compulsive (OBS, 10 items), interpersonal sensitivity (SENS, 9 items), depression (DEP, 13 items), anxiety (ANX, 10 items), anger-hostility (HOS, 6 items), phobic anxiety (PHOB, 7 items), paranoid ideation (PAR, 6 items) and psychoticism (PSYC, 10 items). The final score of the GSI, which represents the average severity score of all the 90 items of the questionnaire, is thought to be a reliable measure of psychological distress. The cut-off score for the GSI used in this study is 0.57, as indicated by the existing literature [16,17]: scores equal to or above 0.57 are considered to be indicative of "dysfunctional" subjects (distressed subjects showing symptoms of somatic and psychological suffering, whose severity lies "within a dysfunctional range"), as opposed to "functional" subjects (healthy subjects, whose symptom severity lies "within a functional range"). "Dysfunctional" subjects have a high probability of psychiatric disorders. The 9 subscale scores represent the average score of positively answered items in each subscale.

### Statistical analysis

Descriptive statistics as means, medians and standard deviations were calculated. The non-parametric Mann-Whitney U test was performed to compare median scores on the SCL-90-R GSI and subscales in patients with IBS only and in patients with both IBS and FD, as well as in IBS patients with PDS and IBS patients with EPS. A *P *value of 0.05 or less was regarded as statistically significant and all reported *P *values were two-sided. The SPSS statistical software (version 13.0) [18] was used for the analyses.

## Results

**Figure 1:**
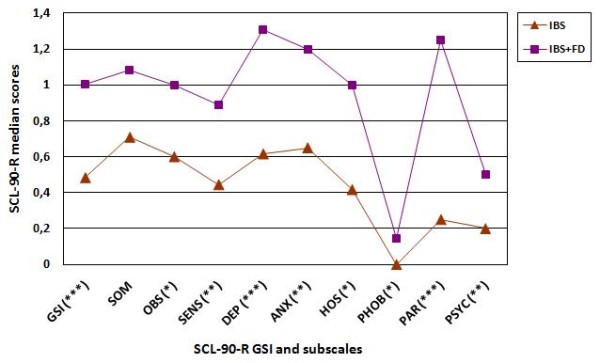
**Line graph comparing median scores of the SCL-90-R GSI and subscales between patients with IBS and patients with IBS and FD**. IBS = irritable bowel syndrome; FD = functional dyspepsia; GSI = global severity index; SOM = somatization; OBS = obsessive-compulsive; SENS = interpersonal sensitivity; DEP = depression; ANX = anxiety; HOS = anger-hostility; PHOB = phobic anxiety; PAR = paranoid ideation; PSYC = psychoticism. (*) = *P *≤ 0.05; (**) = *P *≤ 0.01; (***) = *P *≤ 0.001.

Patients were classified into two groups: patients with IBS only (n = 56, F = 43, mean age 41.6 ± 13.3 years) and patients with both IBS and FD (n = 26, F = 24, mean age 41.8 ± 11.5 years). The main demographic and clinical characteristics of the patients are reported in Table 1. The psychopathological features of the two groups of patients were compared using the SCL-90-R. Patients with IBS and FD showed a significantly higher median score of the GSI than patients with IBS alone (1.00 (interquartile range: 0.68-1.41) vs 0.48 (0.31-0.91), *P *= 0.003) (Figure 1). The group with IBS and FD also scored significantly higher than the group with IBS alone on eight out of the nine SCL-90-R subscales: obsessive-compulsive (1.00 (0.60-1.50) vs 0.60 (0.50-0.97), *P *= 0.03), interpersonal sensitivity (0.89 (0.30-1.55) vs 0.44 (0.13-0.75), *P *= 0.01), depression (1.31 (0.67-1.78) vs 0.61 (0.30-1.00), *P *= 0.000), anxiety (1.20 (0.77-1.60) vs 0.65 (0.32-1.10), *P *= 0.002), anger-hostility (1.00 (0.33-1.33) vs 0.42 (0.16-0.62), *P *= 0.02), phobic anxiety (0.14 (0.00-0.75) vs 0.00 (0.00-0.28), *P *= 0.02), paranoid ideation (1.25 (0.50-2.00) vs 0.25 (0.00-0.95), *P *= 0.000) and psychoticism (0.50 (0.20-1.10) vs 0.20 (0.00-0.40), *P *= 0.002) (Figure 1). The group with IBS and FD continued to report significantly higher SCL-90-R GSI and subscale scores than the group with IBS alone, even after application of a Holm adjustment for multiple comparisons, with the exception of the obsessive-compulsive subscale (adjusted *P *value = 0.06). With regard to the two FD subtypes, of the 26 IBS patients who also fulfilled the criteria for FD, 17 had PDS (65.4%, F = 16, mean age 40.0 ± 12.0 years) and 9 had EPS (34.6%, F = 8, mean age 45.0 ± 11.0 years). No difference was found in the median scores of the SCL-90-R GSI and subscales between IBS patients with PDS and IBS patients with EPS (*P *ranging from 0.07 to 0.97).

**Table 1:** Main demographic and clinical characteristics of patients with IBS and patients with IBS and FD

Disease	IBS		IBS + FD	
	***N***	***%***	***N***	***%***

**Gender**				

Male	13	23.2	2	7.7

Female	43	76.8	24	92.3

**Age**				

< 20	0	0	0	0

20-29	11	19.6	5	19.2

30-39	18	32.1	6	23.1

40-49	11	19.6	8	30.8

50-59	7	12.6	5	19.2

60-69	9	16.1	2	7.7

≥ 70	0	0	0	0

**Type of referral**				

Self-referral	19	33.9	9	34.6

Primary care	30	53.6	15	57.7

Other gastroenterologists	7	12.5	2	7.7

**Total**	56	100	26	100

IBS = irritable bowel syndrome; FD = functional dyspepsia.

## Discussion

IBS and FD are highly prevalent in the general population and they frequently occur simultaneously, with a high percentage of patients satisfying diagnostic criteria for both disorders. The estimated prevalence of FD in IBS patients ranges between 56% and 87% in population-based studies [3,19], while the same analysis performed on gastroenterological outpatients shows percentages ranging between 66% and 87% [5,8,20,21]. On the other hand, dyspeptic subjects match the symptom-based diagnosis of IBS in 14%-29% of cases in community-based settings [22,23] and in 13%-46% of cases in patient-based series [6,24,25]. The high variability of IBS and FD co-occurrence rates across studies may be attributed to differences in the geographical origin of the studied population [5], in the size of the patient sample [5,26] and in the applied diagnostic criteria (standardized Rome criteria versus diagnoses based on clinical opinion; Rome III criteria versus Rome II or Rome I criteria) [5,26].

The frequencies of IBS-FD overlap observed across studies are two to three times higher than those expected from the analysis of the prevalence of each condition in the population. These data point to a relationship between IBS and FD that cannot be explained by the pure chance occurrence of the two disorders, albeit both very common [26], suggesting instead a possible shared pathopyhisiology [5,25]. Overlap between IBS and upper gastrointestinal symptoms can be observed in both FD subtypes, PDS and EPS, with the former showing a higher chance of overlap with IBS than the latter [5].

The present study shows a high frequency of overlap between IBS and FD, equal to 31.7%, supporting previous findings [2,25]. Moreover, it shows no difference in terms of IBS-FD overlap between PDS and EPS, but this result should be interpreted with caution, considering the small sample size of the two subgroups.

IBS and FD are both associated with psychiatric symptoms and disorders [6,7]. It is widely recognized that IBS patients have a higher prevalence of psychopathology (40%-60% of subjects) - notably anxiety, depression, panic, post-traumatic stress and somatization [7,27] - in comparison with patients affected by organic gastrointestinal disorders (< 25% of subjects) and healthy controls (< 20% of subjects) [7,28]. Compared to IBS, data on psychiatric comorbidity in FD are scarce. FD patients, like IBS patients, show a high prevalence of psychopathology (35%-86% of subjects), in particular anxiety, depression, somatization and alexithymia [29-31]. The prevalence rates are higher than those reported for patients affected by organic dyspepsia (25% of subjects) and healthy controls (< 20% of subjects) [32,33]. To our knowledge, there is only one study [34] which has evaluated psychological factors in FD using the Rome III definition and distinguishing between the two FD subtypes: this study has found that anxiety is linked to PDS, but not to EPS, and that depression is not linked to either of them.

There appear to be similarities in the frequency and type of psychiatric comorbidity in IBS and FD [35], but psychological factors are not believed to be an essential underlying cause for either of them: in accordance with the biopsychosocial model of functional gastrointestinal disorders [36], psychological factors, through their interaction with biological and social factors, influence the clinical expression and outcome of these disorders [37], thus determining health-care services utilization [11] and health-related quality of life [7].

It is not well understood whether the overlap between lower and upper gastrointestinal symptoms in IBS patients, which is known to increase the overall gastrointestinal symptom severity [25], is also associated with an increased level of psychopathology. In recent studies on IBS patients, coexisting upper gastrointestinal symptoms, compatible with the diagnosis of FD, seem to increase the level of psychopathology [8,9]. A study by Talley *et al*. [8] reports a statistically significant positive correlation between the SCL-90-R GSI and the number of gastrointestinal symptoms in IBS patients with upper gastrointestinal symptoms. A subsequent study by Balboa *et al*. [9] shows that the presence of FD in IBS patients has a significant negative impact on the psychological status. On the other hand, a recent study by Mikocka-Walus *et al*. [10] has found that the number of comorbid functional gastrointestinal disorders does not correlate with higher scores for anxiety and depression.

The present study shows that patients meeting the Rome III diagnostic criteria for both IBS and FD have a greater severity of psychopathological symptoms than those with IBS only, rating significantly higher on the GSI and on eight out of the nine SCL-90-R subscales: obsessive-compulsive, interpersonal sensitivity, depression, anxiety, anger-hostility, phobic anxiety, paranoid ideation and psychoticism. Moreover, only the group of patients with both IBS and FD presents a median score of the GSI > 0.57, the cut-off point above which patients are considered "dysfunctional" (i.e. with a high probability of psychiatric disorders) [16,17]. The results of this study are in accordance with those obtained by Talley *et al*. [8] and Balboa *et al*. [9], which used the Rome II diagnostic criteria. The present study applied the Rome III criteria and assessed the association of psychopathological features with IBS-FD overlap, differently from Mikocka-Walus *et al*. [10], who have not investigated patients with IBS-FD overlap, but rather a small size population with any type of functional gastrointestinal disorder.

The findings of this study are not surprising: given the strong link between IBS, FD, and psychiatric comorbidity, it seems likely that patients with more gastrointestinal symptoms have also more - or more severe - psychiatric symptoms. The higher prevalence of hypersensitivity to gastric distension observed in patients with both IBS and FD in comparison to patients with IBS or FD only [25] could be a pathophysiological mechanism capable of explaining the difference not only in the severity of gastrointestinal symptoms, but also, hypothetically, in the severity of the psychological suffering.

It is not known whether this effect is different between IBS patients with PDS and IBS patients with EPS. According to recent studies, FD appears to include different types of patients with distinct underlying pathophysiologies who require different management [2,12]. Since functional gastrointestinal disorders are viewed as multifactorial disorders in which physiological, psychological and social factors are strictly intertwined [7], different FD subtypes may differ not only in pathophysiology, but also in psychopathology. As a preliminary analysis, the present study compared psychopathological features between IBS patients with PDS and IBS patients with EPS, showing that these two subgroups of patients are characterized by comparable scores on the SCL-90-R GSI and subscales. However, given the small size of the two subgroups, the lack of significant findings may be due to a lack of statistical power to identify differences: these findings have to be considered provisional and should be confirmed in a larger population sample. For this reason, we are testing these observations in a new study in which we are increasing the sample size.

In the present study, 11.0% of patients were referred from other gastroenterologists. It could be argued that these patients may behave differently from primary care- or self-referred patients, but we found that they did not differ by any demographic or psychological variable. In any case, some bias cannot be ruled out and our third level referral centre sample may not accurately represent the entire population of patients with IBS or with IBS and FD.

To conclude, in clinical practice the higher levels of psychopathology observed in IBS patients with FD in comparison to those without FD may result in an increased medical consultation and in a greater impairment of health-related quality of life, with significant socio-economic implications. According to Hu *et al*. [38], coexisting depression and anxiety act as catalysts for IBS patients to seek medical care, thus increasing the socio-economic burden of this functional gastrointestinal disorder. The presence of FD in IBS patients, by causing more severe depressive and anxiety symptoms, may exacerbate this effect.

## Conclusions

Approximately one-third of IBS patients recruited in this study fulfill the Rome III criteria for FD. IBS patients with FD show significantly higher scores on the SCL-90-R GSI and subscales than IBS patients without FD, supporting the hypothesis that the presence of FD in addition to IBS is associated with an increased severity of psychopathological features. These findings suggest that a substantial subset of patients of a third level gastroenterological referral centre presenting both IBS and FD symptoms may benefit from psychological assessment and treatment. Appropriate assessment and treatment are likely to have a positive impact on patients' well-being and health care utilization.

## Competing interests

The authors declare that they have no competing interests.

## Authors' contributions

DP, RC, MA and ESC contributed equally to this work; NP, DB, MB and ESC designed research; DP analyzed data; DP, RC and ESC wrote the paper; MB and ESC revised the manuscript. All authors read and approved the final manuscript.

## Pre-publication history

The pre-publication history for this paper can be accessed here:

http://www.biomedcentral.com/1471-230X/11/94/prepub
